# Hollow glass microspheres/phenolic syntactic foams with excellent mechanical and thermal insulate performance

**DOI:** 10.3389/fchem.2023.1216706

**Published:** 2023-06-01

**Authors:** Hui Wang, Rui Yan, Hua Cheng, Mingmin Zou, Hua Wang, Kang Zheng

**Affiliations:** ^1^ Department of Chemistry and Chemical Engineering, Hefei Normal University, Hefei, China; ^2^ Institute of Solid State Physics, Hefei Institutes of Physical Science, Chinese Academy of Sciences, Hefei, China; ^3^ Information Materials and Intelligent Sensing Laboratory of Anhui Province, Anhui University, Hefei, Anhui, China

**Keywords:** syntactic foam, phenolic resin, microspheres, thermal insulate properties, mechanical properties

## Abstract

Syntactic foams with low density as well as low thermal conduction and proper mechanical performance are vitally important for aerospace, marine, and automotive industries. Here, phenolic-based syntactic foams were fabricated by combining the hollow glass microsphere (GMs) with phenolic resin of *in situ* synthesis. Benefited from the stirring and hot-pressing treatment, microspheres dispersed homogeneously in the resin matrix and it greatly reduced the density of the composites. Stretching and compression tests were performed to investigate the mechanical behavior of the foams. It is found that both the compressive and tensile strength decreased as the filler loadings increasing. While the elasticity modulus was improved. On the other hand, thermal properties tests revealed superior thermal stability and thermal insulate performance of the composites. The final residue content of the synthetic foam with 40 wt% filler was improved by ∼31.5% than that of the neat one at 700°C. And samples with 20 wt% microspheres reached a minimum thermal conductivity value of approximately 0.129 W (m·K)^−1^ which is ∼46.7% lower than that of neat resin [0.298 W (m·K)^−1^]. This work provides a feasible strategy to construct syntactic foams with low density and ideal thermal properties.

## 1 Introduction

Syntactic foam is a type of porous material by mixing hollow particles (the filler) with a resin system (the binder) (Klempener and Sendijarevic, 2004; Devi et al., 2007). This kind of syntactic foam can also be defined as a special type of particulate-filled polymer composite for it is prepared by adding a lower density filler into the binder matrix. The hollow particles can be consisted of inorganic materials, such as glass and silica, or polymeric materials, such as epoxy, unsaturated polyester, phenolics, and polyvinyl chloride (Balch and Dunand, 2006; Yin et al., 2016). Thermoset resins are often chosen as the binder, such as epoxy, phenolic, or polyester (Narkis et al., 1982; Karthikeyan et al., 2000; Balch and Dunand, 2006; John and Nair, 2022). A lot of voids were produced by introducing the hollow particles like a porous material with closed cells and that may be why we call it syntactic foam. Compared to normal cellular foams, they show advantages such as high specific strength, sound absorption, and thermal properties (Narkis et al., 1982; Porfiri and Gupta, 2009; Cya et al., 2021). Therefore, syntactic foams are widely used in engineering applications, such as aerospace, marine, automotive, and building industries (Shunmugasamy et al., 2012; Jiang et al., 2014; Zhao et al., 2015; Song et al., 2021). This kind of polymeric foams is usually applied for thermal insulation due to its excellent thermal resistance, which can save energy for buildings efficiently (Latha et al., 2015; Moradibistouni et al., 2020). They can also be used as core material of sandwich panels for structural applications, for example, building floors and roof structures (Manalo et al., 2016). Compared with traditional core materials (such as wood and metal honeycomb) polymeric foams show advantages of low cost, low thermal conductivity as well as proper mechanical properties (Mazzuca et al., 2021). What’s more, their mechanical and thermal properties can be tailored by designing the cell structure of the foams, thus different lightweight multi-functional syntactic foams can be obtained (Gibson and Ashby, 1997).

Generally, different types of syntactic foams with various mechanical properties can be created by using different kinds of binders or microspheres, adding different amounts of hollow filler particles, modifying the interface between the fillers and the binders, and modulating the overall foam density (Huang et al., 2006; Kumar and Ahmed, 2016). Wouterson and his co-workers investigated the influence of different compositions of syntactic foam on its mechanical and fracture properties (Wouterson et al., 2007). Gupta et al. characterized syntactic foams for flatwise (specimen aspect ratio of 0.5) properties and studied the effect of change in the internal radius of cenospheres. The radius ratio range is from ∼0.863 to ∼0.922 (Nikhil et al., 2004). Study on phenolic hollow microspheres (PHMS)-filled vinyl ester (VE) composites has also been carried out (Yusriah and Mariatti, 2013). However, these syntactic foams are flammable due to their organic nature. And they undergo thermal decomposition, releasing toxic smoke or gases at even 200–300°C. The mechanical performance of these foams was serious damaged when they were subjected to elevated temperatures or fire. All these drawbacks mentioned above greatly limited the application of syntactic foams (McKenna and Hull, 2016; Mazzuca et al., 2022). Considerable attempts have been made by researchers to develop high-performance syntactic foams.

Phenolic resin and phenolic resin foam have drawn much attentions in recent years due to their good mechanical properties, heat resistance, dimensional stability, and chemical resistance (Ozkutlu et al., 2018; Yuan et al., 2020). There are carbon-carbon bonds in the aromatic ring of the phenolic structure which will slowly break up to release carbon as subjected to thermal stress. So, phenolic resin and phenolic-resin-based foam give off little smoke upon ignition and exhibit high char yield (John, 2010). The most glaring omission is their undesirable friable nature. However, it can be significantly reduced by introducing proper fillers. Our group have synthesized phenolic resin/attapulgite (AT) nanocomposites by the *in-situ* polymerization and explored the influence of AT on the mechanical properties of the system (Wang et al., 2015). As a common thermosetting resin, phenolic resin has been used as a polymeric adhesive due to its excellent bonding strength (Maldas et al., 2012). The preparation of phenolic syntactic foam mainly depends on this physical nature of the resin. And it can be either in the form of a liquid or solid. Researchers have explored various kinds of phenolic resins for the processing of syntactic foams. Ma et al. have studied the effect of coupling agent on mechanical properties of hollow carbon microsphere based syntactic foam and found that better interfacial adhesion could be induced from coupling agent treated microspheres, hence resulted in better mechanical properties (Zhang and Ma, 2010). Recently, Karbalaei-Bagher M et al. have discussed curing characteristics of phenolic syntactic foams based on oxygen plasma treated microspheres (Karbalaei-Bagher et al., 2020). Researchers also developed phenolic syntactic foam core sandwich composites and investigated their thermal stability, minimum concentration of oxygen required to catch the fire and flammable characteristics (SJ Amith et al., 2020). And the results revealed that the phenolic syntactic foam core was thermally more stable than glass/epoxy face skins up to 450°C. The minimum concentration of oxygen required for burning was found to be 30%, in which phenolic syntactic foam core helped in flame isolation, whereas glass/epoxy face skins contributed to flame spread in the event of burning of sandwich composites (SJ Amith et al., 2020).

However, resin’s viscosity has a great influence on the construction of phenolic syntactic foams. If the viscosity is very low, it can be mixed with fillers easily. If the viscosity is very high, the resin must be dissolved in proper solvent first and the fillers are impregnated with the prepared solution. One significant drawback of this method is the removal of solvent before curing. Moreover, it would need additional chemical agents, which means higher cost and more environmental pollution. In this study we reported a simple and effective way to synthesize a kind of syntactic foams choosing hollow glass microspheres (GMs) as the filler and phenolic resin as the binder. The suitable viscosity of the resin was obtained by *in-situ* polymerization. GMs are fillers commonly used in syntactic foams for their good chemical stability, light weight, excellent mechanical, and thermal physical properties (Kiran et al., 2022). Coated microspheres by phenolic resin were transferred to a metal mould and compressing moulded at 180°C with pressure of 4 MPa for 60 min. Both the mechanical and thermal properties of the syntactic foam were detected and analyzed. A lightweight syntactic foam with good thermal properties and proper mechanical properties were obtained. With integrating these remarkable features in one material, this phenolic syntactic foam is ideal candidates for various applications such as fire-resistant panels, insulation panels for construction industry, low density ablative for space applications and so on.

## 2 Experimental section

### 2.1 Materials

The syntactic foams for this research were fabricated by dispersion of a kind of hollow microspheres in phenolic resins which were synthesized independently. These microspheres were provided by Bengbu Institute of Glass Industrial Design and Research and its properties were listed in Table 1. Formaldehyde solution (37 wt%) and phenol used as the main raw material were supplied by Sinopharm Chemical Reagent Co., Ltd. (Shanghai, China) and Shanghai Titan Scientific Co., Ltd. (Shanghai, China), respectively. Sodium hydrate acted as catalyst while paratoluenesulfonic acid sodium was used as curing agent. They were also purchased from Sinopharm Chemical Reagent Co., Ltd. and Shanghai Titan Scientific Co., Ltd., respectively. All the reagents mentioned above were analytic grade and used without any purification.

**TABLE 1:** Physical properties of the hollow glass microspheres (GMs).

Sample	True density (g·cm^−3^)	Packing density (g·cm^−3^)	Static pressure (MPa)	Diameter (um)
GMs	0.3–0.6	0.1–0.3	10–60	30–100

### 2.2 Synthesis of the syntactic foams

The phenol(P) and formaldehyde(F) were mixed in a 500 mL three-necked round bottom flask with a mole ratio of P/F = 1:2. An aqueous solution of NaOH-20% was added into the mixture. The reactions were carried out at 80°C for 4 h with a stirrer, a water bath heating unit, and a reflux condensing tube. After cooling to room temperature, acetic acid solution (36–37 wt%) was added into the reactor. Then the reaction mixtures were left under a vacuum of 0.075 MP at 75°C to remove the by-products such as water and excess formaldehyde. Then we got the phenolic resins used as the binder.

**FIGURE 1:**
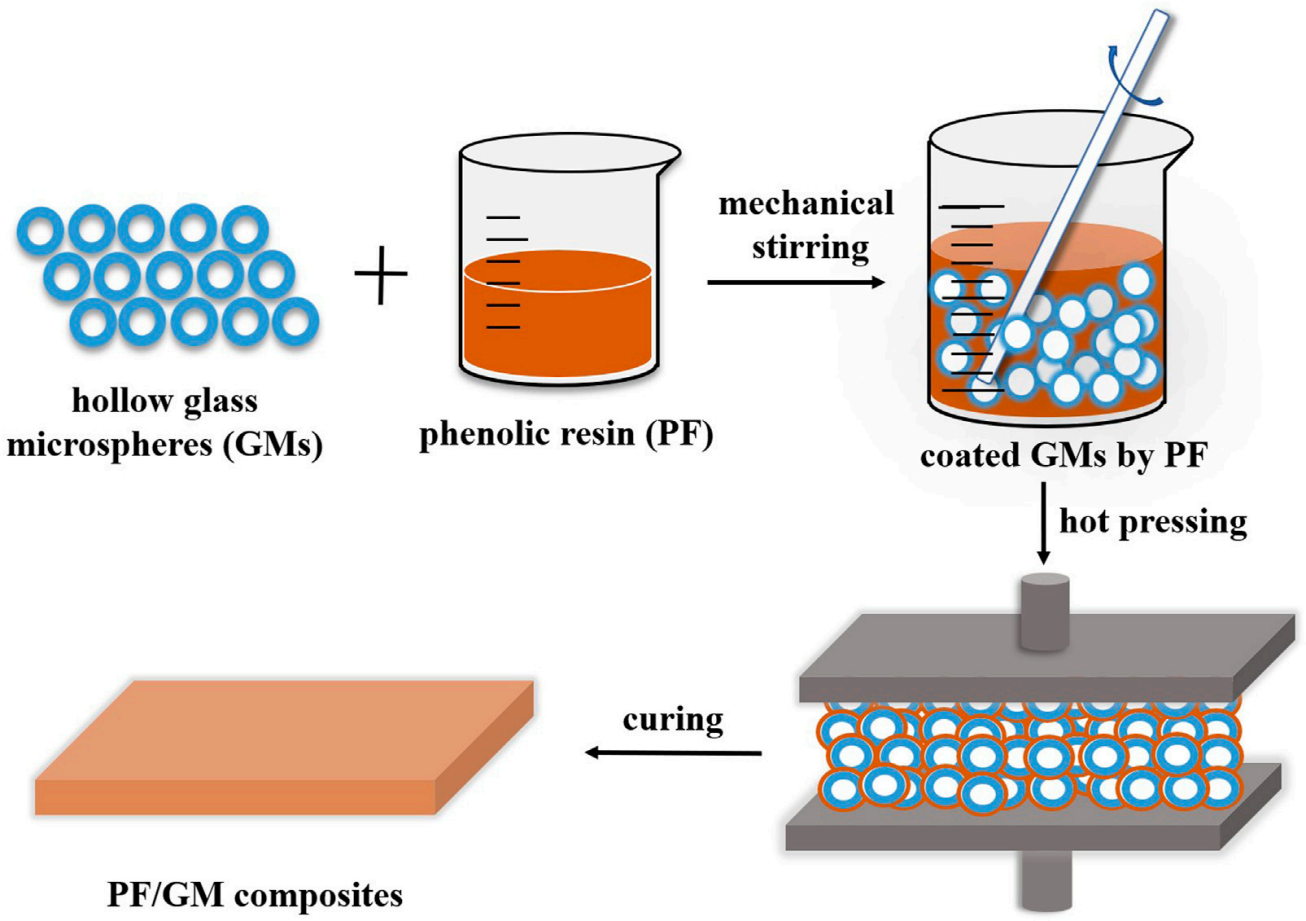
Schematic of the fabrication of PF/GM syntactic foams.

Schematic representation of the syntactic foams was summarized in Figure 1. The microspheres were added to the phenolic resins in multiple steps to avoid agglomeration. Because of the lower density of the microspheres than the binder, the microspheres tend to float to the top surface. This effect was minimized by continuous stirring and injecting the mixtures in a proper mold within a short time, followed by hot compaction for 60 min at 180°C 4 MPa. Then the mold was left in a drying oven to cure for 10–15 h at 150°C. Finally, we took the mold out and cooled it to room temperature. By adding different amounts of fillers to the matrix, syntactic foams with various mass fractions of microspheres (PF/GMs) were thus obtained, named as PF/GM-10wt%, PF/GM-20wt%, PF/GM-30wt%, PF/GM-40wt% composites, respectively. Neat resin plates (PF/GM-0wt%) were fabricated according to the same processing conditions for comparison.

### 2.3 Characterization

The analyses of fracture morphology were carried out on a FEI Sirion 200 field emission scanning electron microscope (FE-SEM) (FEI company, Hillsboro, OR, United States) at 10 kV. The syntactic foam samples were cut into small pieces and coated with gold to prevent charge buildup.

Thermogravimetric analyses (TGA) were performed with a PerkinElmer Pyris-1 TGA (PerkinElmer Inc., Shelton, CT, United States). The samples of pure PF resins and the syntactic foams were heated from 50°C to 700°C at a heating rate of 10°C min^−1^ under nitrogen atmosphere.

In order to investigate the heat-transfer properties of the composites we measured the thermal conductivity of the neat resins and the syntactic foams. The thermal diffusivity, λ, was determined by the laser fiash method (Netzsch, LFA-457) at 25°C. The specific heat C_p_ was measured using differential scanning calorimetry (PerkinElmer, Diamond DSC). The resulting thermal conductivity κ was calculated from the measured thermal diffusivity λ, specific heat C_p_, and density ρ according to the equation (Eq. 1):

$$\kappa =\lambda \rho C_{p}.$$ (1)

The mechanical tests of tension and compression were performed with different shapes of the foams on a universal testing machine (model CMT4204, SANS Test Machine Co., Ltd., Shenzhen, China). The results presented are an average of five tests. The tensile testing was carried out according to ISO 527:1997 (GB/T1040-2008). The test samples were made into rectangular splines with a length of 100 mm, width of 10 mm and thickness of 4 mm. They were loaded between two stainless steel clamps at a cross-head speed of 2 mm·min^−1^ at room temperature.

The compression test was carried out in accordance with ISO 604:2002 (GB/T1041-2008) at room temperature. The syntactic foams were machined to blocks of 25 mm^3^ × 25 mm^3^ × 25 mm^3^. The samples were compressed between two stainless steel plates with the cross-head speed of 2 mm·min^−1^, and the foams were pressed up to 10 mm deflection. The compression modulus, Ec, was calculated by the equation (Eq. 2) (Wouterson et al., 2005):

$$E_{C}=\frac{mt}{A}$$ (2)

where m is the slope of the initial linear region of the load–deflection curve, and A is the cross-sectional area, and t is the thickness of the syntactic foam.

## 3 Results and discussion

### 3.1 The density of the samples

**FIGURE 2:**
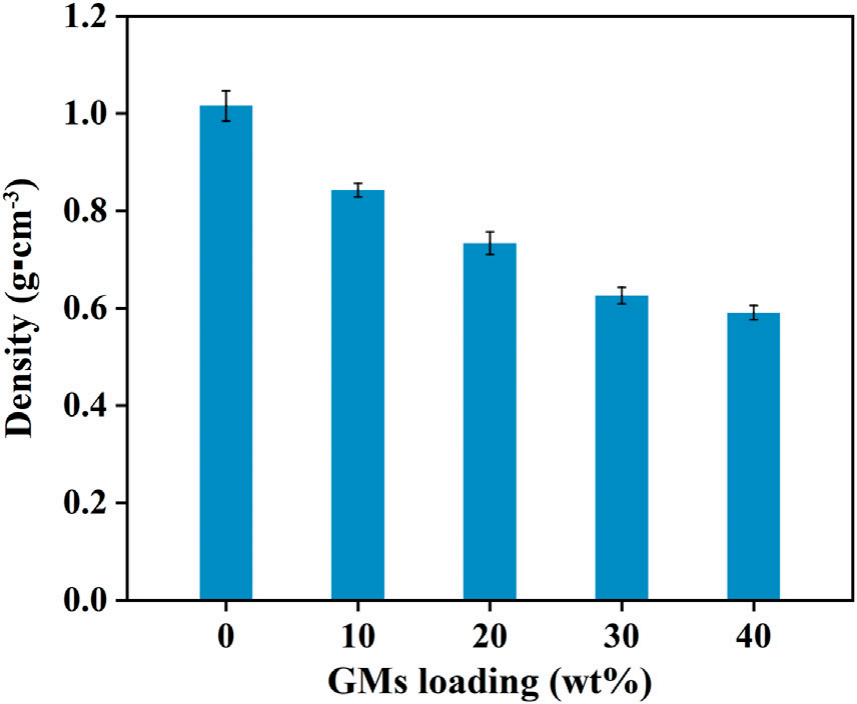
The densities of syntactic foams with various microsphere loadings.

**FIGURE 3:**
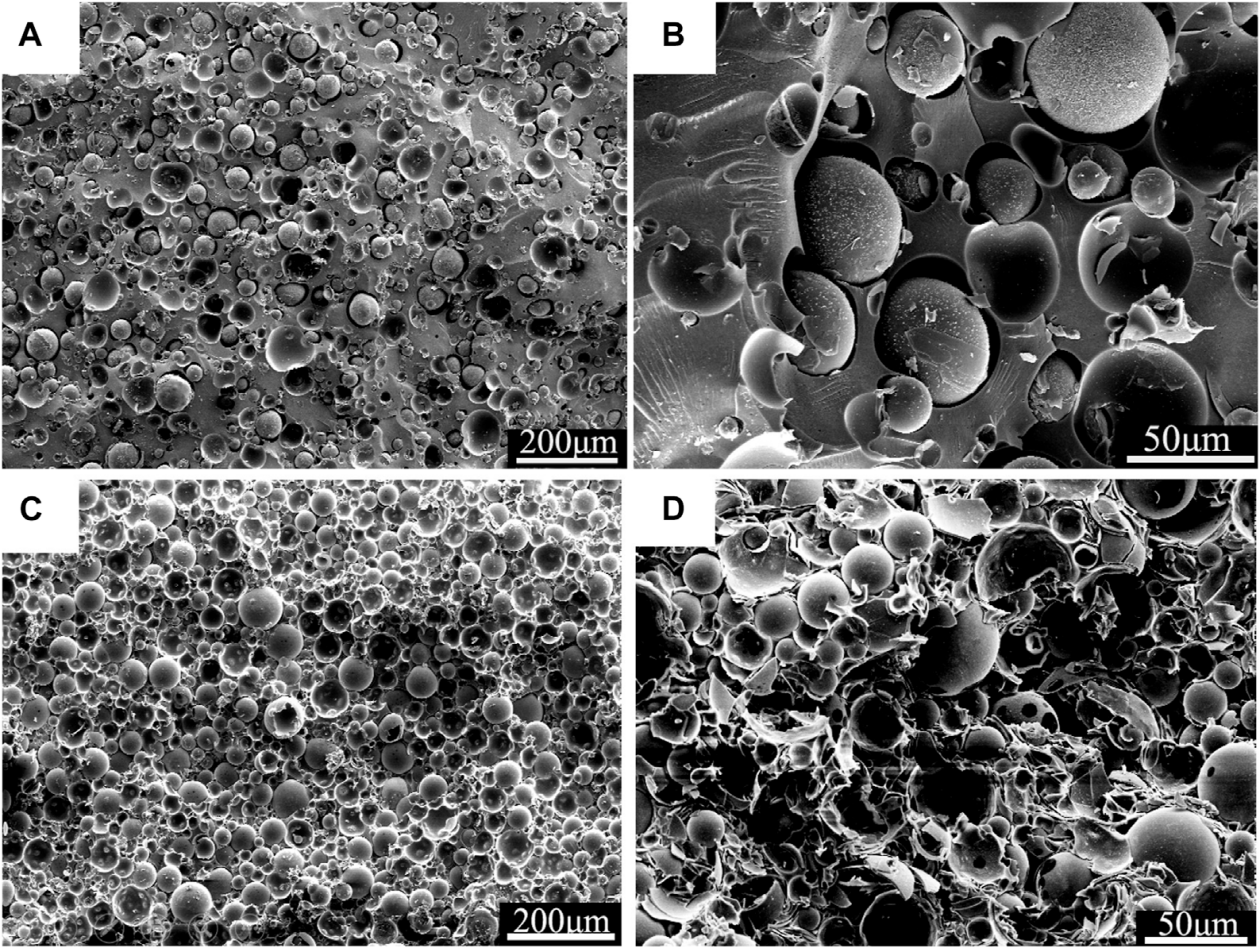
SEM images for PF/GM-10wt% **(A)**; The fracture surface of PF/GM-10wt%. After tensile test **(B)**; SEM images for PF/GM-30wt% **(C)**; SEM micrograph for badly crushed syntactic foam with 40wt% GMs **(D)**.

Figure 2 presented the experimental densities of the neat resin and syntactic foams. It is clear that syntactic foams showed much lower density than that of the neat resin plate. Moreover, the densities decreased with the increasing of microsphere content. Scanning electron microscope (SEM) photomicrographs in Figures 3A–D confirmed the existence of microspheres that homogeneously dispersed in the resin matrix. The glass hollow microsphere is a kind of lightweight materials, so it is quite understandable that the density of the syntactic foams decreased with the increasing of microsphere content. However, it should be noted that densities of the samples cannot be reduced indefinitely because the resin’s capacity for wetting is limited. What’s more, as we added too much glass microspheres, they began to crush as a result of being squeezed by each other during the process of hot compaction. It is clear to see badly crushed microspheres in the matrix everywhere (Figure 3D).

The experimental densities of the samples were obtained from dividing the mass by the volume. Here, the volume includes not only the matrix and microballoons but also some voids existed in the matrix (Figure 3B). Because of the existed voids, experimental densities are lower than the theoretical densities. The voids in the matrix will affect the mechanical properties of the syntactic foams. However, these voids are conducive to heat insulation. The detailed numerical results of the theoretical densities have little impact on subsequent discussions. Thus, we did not further test the theoretical densities of the samples. Generally, being lightweight means material and energy savings. So, it is a key technological requirement for developing materials especially used in the areas of aircraft, spacecraft, and automobiles.

### 3.2 Tensile test

**FIGURE 4:**
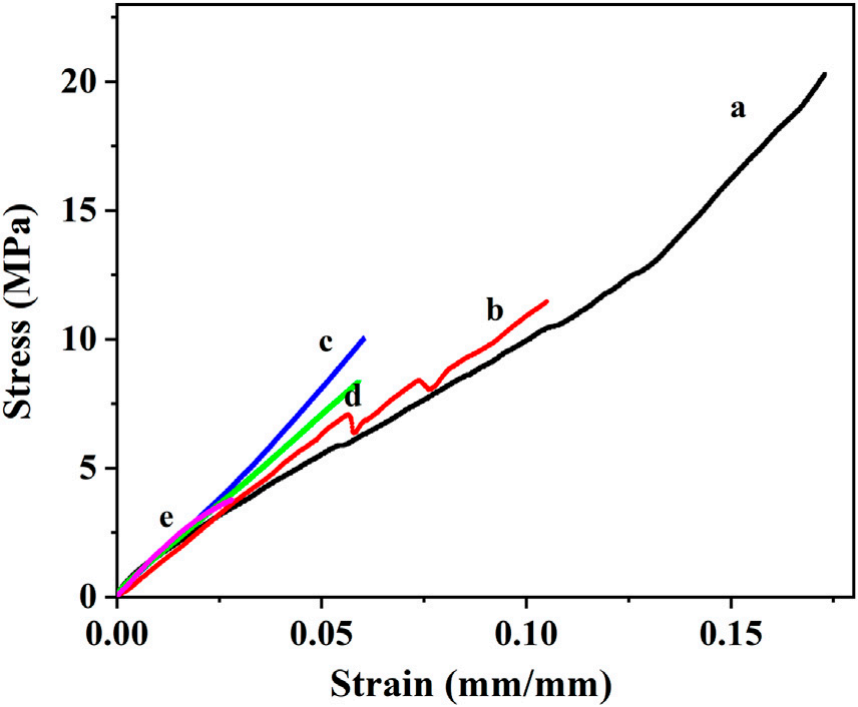
The stress-strain curve of the tensile experiment. (a) Neat phenolic resins, (b) PF/GM-10wt%, (c) PF/GM-20wt%, (d) PF/GM-30wt%, (e) PF/GM-40wt%.

**FIGURE 5:**
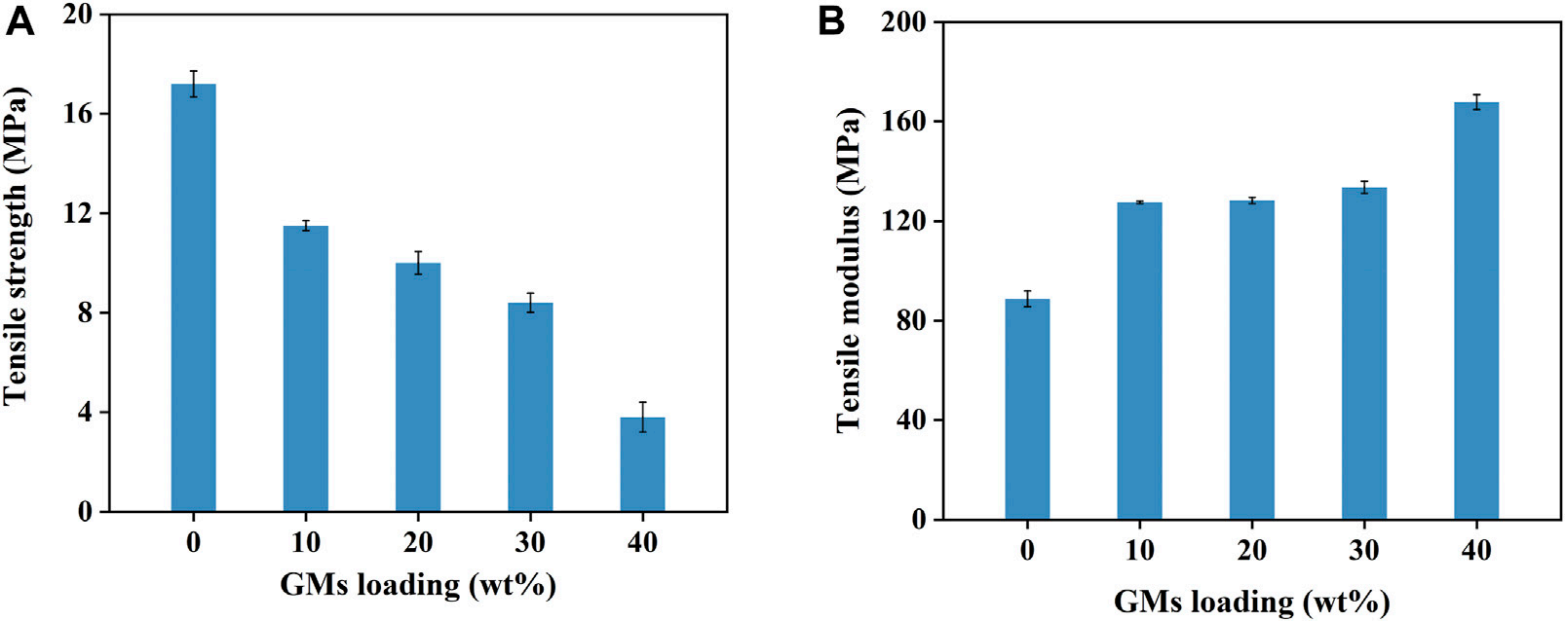
Tensile strength **(A)** and tensile modulus **(B)** of the neat resin and syntactic foams.

Figure 4 shows the stress-strain curves of the tensile experiment for neat resin and syntactic foam with various contents of microspheres. The tensile strength decreased with the increasing of microsphere loading, while the corresponding tensile modulus increased (Figure 5). There were many researches on the influence of the increasing content of hollow microspheres on tensile modulus of the composites (Bardella and Genna, 2001; El-Hadek and Tippur, 2002; Fine et al., 2003). They concluded that the trend of variation of tensile modulus largely depends on the type of microspheres used. Figure 3B shows a typical SEM fractograph of a tensile specimen with 10wt% hollow glass microspheres. We can see some voids existed among the matrix which will affect the mechanical properties. Luxmoore and Owen concluded that a crack will initiate from an oversized void when a composite is subjected to tensile loading (Luxmoore and Owen, 1978). As a matter of fact, almost all the specimens fractured at a cross-section containing voids. Luxmoore and Owen suggested that the failure of the foam is attributed to the failure of the resin matrix (Luxmoore and Owen, 1978). Introduction of microspheres reduces the resin fraction and increases the inhomogeneity content which leads to poor interfacial strength between the binder and the filler, consequently reducing the tensile strength of the composites. The authors assumed that the matrix served as the load-bearing phase in the composite whereas the hollow microspheres only provided light weight and minimal strengthening effect.

We can see a rough surface of the phenolic resin after brittle fracture and a lot of folded structures were recognized in Figure 3B indicating the plastic yielding of the resin. Apart from that, integral microspheres can be observed on the fracture surface, which demonstrated that when the composites broke under a tensile load the microspheres retained intact. All these facts confirmed the role of the resin matrix serving as the load bearing in the failure of syntactic foam under tensile loading. This conclusion is also in accordance with the results of Luxmoore and Owen’s. Furthermore, it was obvious to see some footprints left by the microspheres, which suggested the presence of filler debonding accompanied with the crack growing over the interface between the resin and the microspheres.

### 3.3 Compression test

**FIGURE 6:**
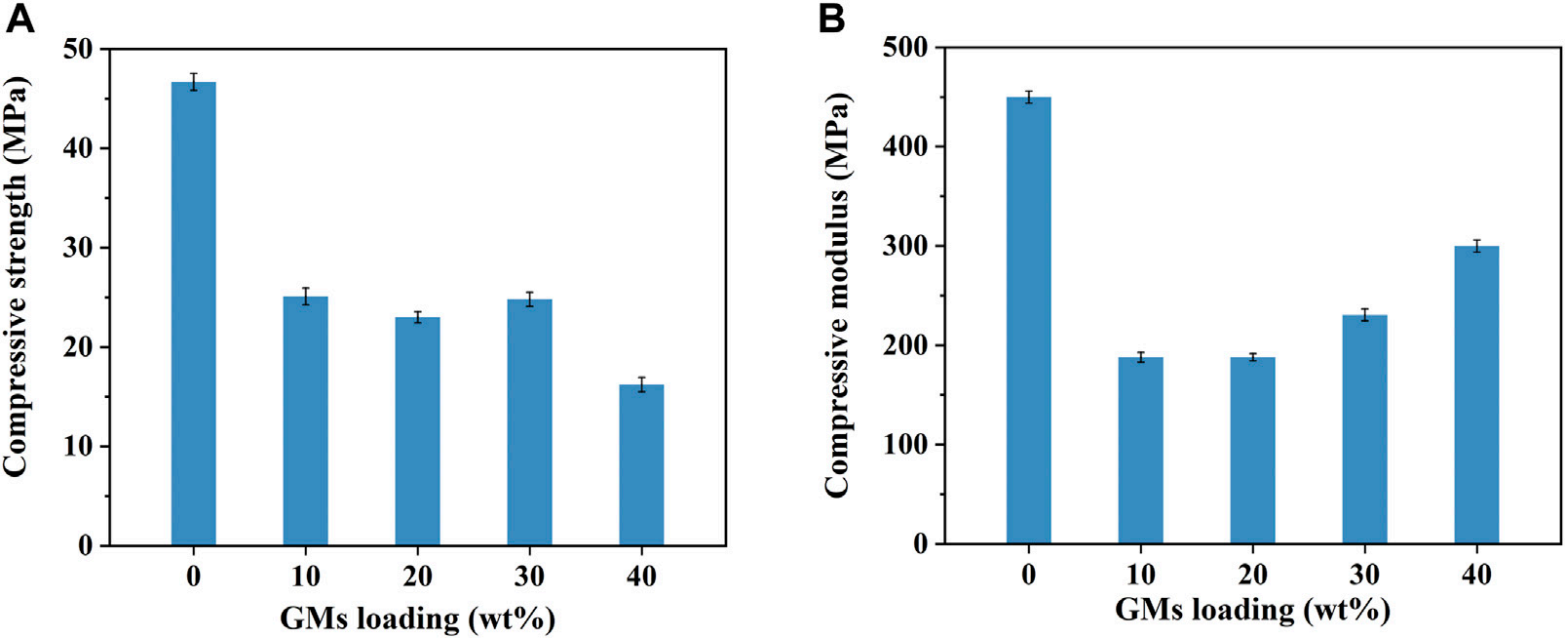
Compressive strength **(A)** and modulus **(B)** of the neat resin and syntactic foams.

**FIGURE 7:**
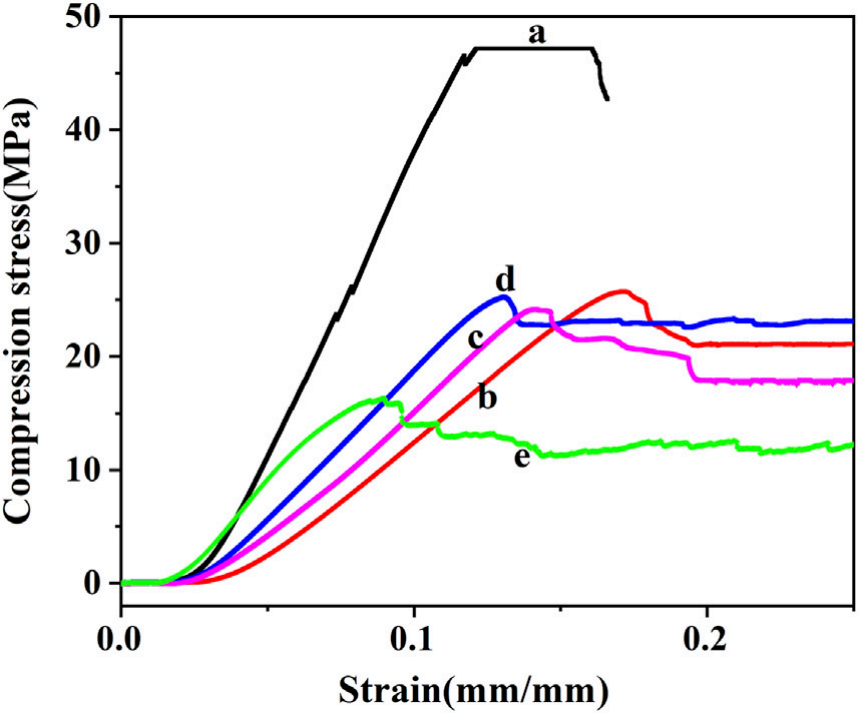
Compression stress–strain curves of neat resin and syntactic foam with various contents of microspheres. (a) Neat phenolic resins, (b) PF/GM-10wt%, (c) PF/GM-20wt%, (d) PF/GM-30wt%, (e) PF/GM-40wt%.

The detail results of compression tests are summarized in Figure 6. Introduction of hollow microspheres leads to the decrease of compression strength (Figure 6A). What’s more, compared with the neat resin, the compression stress of composites with 40wt% microspheres decreased significantly. While the strength and modulus of other foams (with 10wt%, 20wt%, 30wt% microspheres) are similar to each other (Figure 6A, B). The microspheres act as voids and weaken the structure (Wouterson et al., 2007). Figure 7 shows the plots of compression stress–strain curves of neat resin and syntactic foam with various contents of microspheres. As shown in Figure 7 each individual curve exhibits a multistage deformation response when exposed to compressive loading. The initial linear region corresponds to the elastic behavior of the foam. At the end of the linear region a yield point is reached and the load becomes nearly constant followed by a slight decrease, which is called plateau region.

**FIGURE 8:**
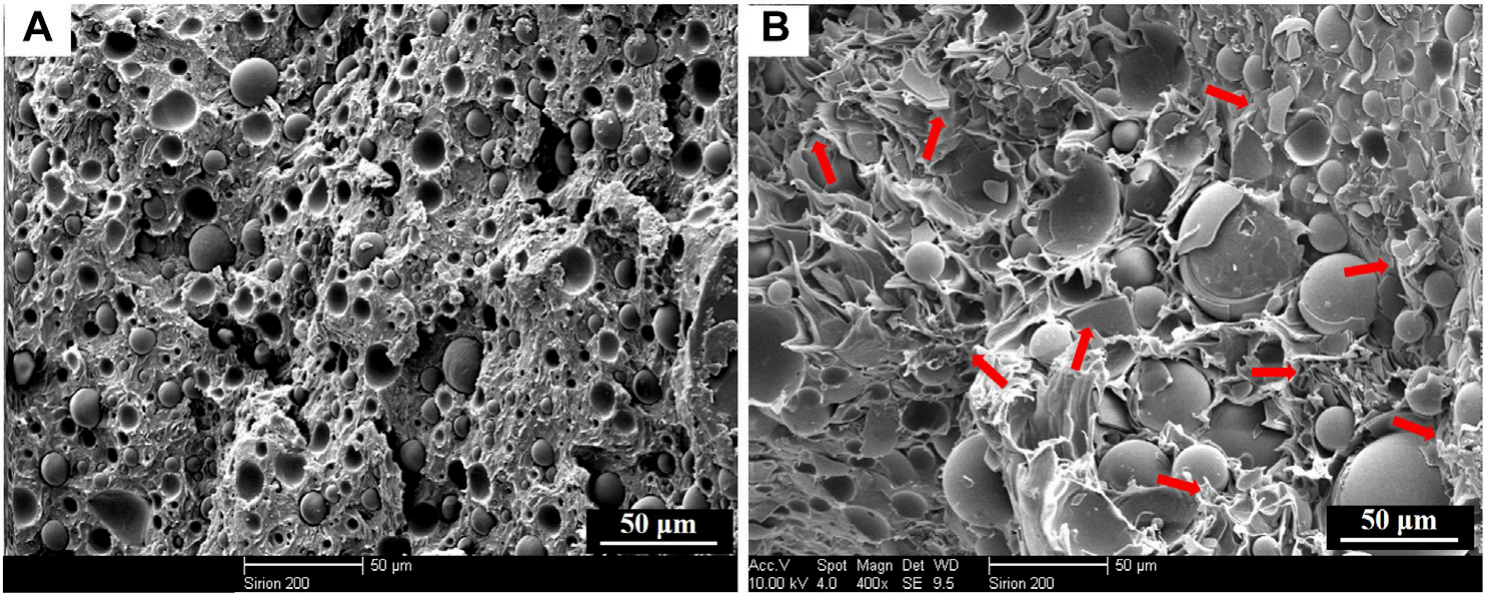
SEM images for PF/GM-20wt% **(A)**; The fracture surface of PF/GM-20wt% after compression test **(B)**.

This plateau region attributes to the energy absorption during the process of microballoons crushing (Gupta et al., 2001). To better understand the function of microspheres for compression plateau region, we analyze cross section map of the tested foams (Figure 8). Compared with that of the sample before test, fragments of microspheres are everywhere in the section of the tested foam (marked by red arrows). Glass microspheres are inorganic and rigid. When compressed to a certain extent, they can play a supporting role in the system, which is vital for structural applications. However, when too much microballoons get crushed, further increasing contents result in a gentle downward of the curve. Similar observations were reported by Huang et al. (2010), which is much different from other reports (Goni et al., 1993; Wouterson et al., 2007). This is because that when the strain exceeded a limit value the corners and edges of sample fracture seriously and stress concentrates at void tips. Cracks initiate at these sites and propagate either along microsphere surfaces or through the microspheres (Huang et al., 2010).

### 3.4 TGA characterization

**FIGURE 9:**
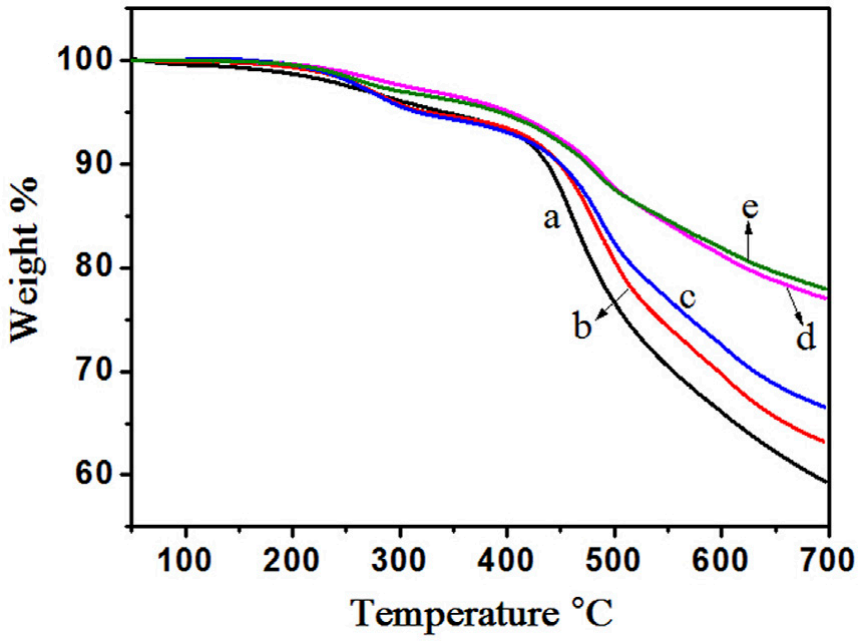
TGA curves for neat resin and synthetic foams (a) Neat phenolic resins, (b) PF/GM-10wt%, (c) PF/GM-20wt%, (d) PF/GM-30wt%, (e) PF/GM-40wt%.

In order to investigate the effect of the microspheres on the thermal stability of foams, thermogravimetric analyses (TGA) were performed and the results were shown in Figure 9. For each individual curve, two apparent stages of weight loss can be observed. The first stage (at about 300°C) is the result of the evolution of some small molecules in the end of the chain (such as carboxyl hydroxymethyl, *etc.*). The second stage is attributed to the major structural (mainly methylene in the resins) decomposition of phenolic resins, which occurs at about 450°C. We compared the temperatures (T_10_) of which the sample weight loss is 10% and found it shift to higher value with the increasing of microsphere content. The results are listed in Table 2. As shown in Table 2, the final residue content of the synthetic foam at 700°C is higher than that of the neat one. Hollow glass microsphere is a kind of chemical stable materials. So, when heated the microsphere it hardly loosed any weight. Introduction of microspheres reduces the continuity of matrix (Wei et al., 2009), consequently improving the thermal stability as result of hindering heat transmission by the hollow glass microsphere which has excellent heat resistance and heat insulation properties.

**TABLE 2:** Thermogravimetric analysis results of neat resin and synthetic foams.

Sample	T_10_/°C	Temperature change/°C	Char residue/% (T = 700°C)	Percent different/%
Neat resin	436.9	—	59.3	—
PF/GM-10wt%	448.5	11.6	63.3	6.7
PF/GM-20wt%	450.1	13.2	66.6	12.3
PF/GM-30wt%	475.2	38.3	77.1	30.0
PF/GM-40wt%	478.9	42.0	78.0	31.5

### 3.5 Thermal conductivity testing

**FIGURE 10:**
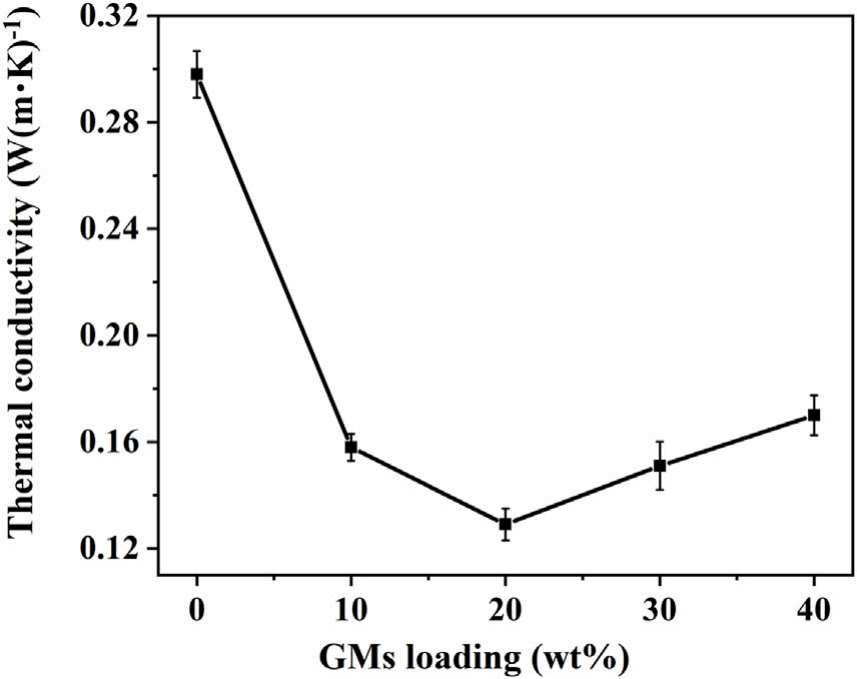
The variation plot of thermal conductivities for the neat resin and synthetic foams.

Table 3 lists the measured thermal conductivities for the neat resin and the composites. Figure 10 displays the variation of these results graphically. The thermal conductivity of the synthetic foam decreased with increase of hollow glass microspheres, and the thermal conductivity reached a minimum value of approximately 0.129 W (m·K)^−1^ when 20 wt% of microspheres were added. Generally, there are two ways for heat transferring in materials with voids: heat radiation and heat conduction. The porosity of the foam was improved as the amount of hollow glass microspheres increased, which made the efficient heat transfer surface per unit area decrease. This is equivalent to increasing the thermal resistance of the synthetic foam, thus reducing the efficiency of heat conducting. Then the heat transfer in per unit area was reduced, consequentially reducing the overall thermal conductivity of the material.

**TABLE 3:** The percent changes of thermal conductivity for neat resin and synthetic foams.

Sample	Thermal conductivity [W (m·K)^−1^]	Percent change (%)
Neat resin	0.298	—
PF/GM-10wt%	0.158	−47.0
PF/GM-20wt%	0.129	−56.7
PF/GM-30wt%	0.151	−49.3
PF/GM-40wt%	0.170	−43.0

However, we observed an increasing of thermal conductivity for PF/GM-30wt% and PF/GM-40wt%. This could be explained by the crushed microspheres. The microspheres dispersed more closely in the resin with the increasing contents as shown in Figure 3C. Then microspheres began to crush as a result of being squeezed by each other in the process of hot compaction, consequently creating effective thermal paths between adjacent microsphere fragments (Figure 3D) embedded in matrix (Kim et al., 2007). So, the thermal conductivity was improved rather than continuously decreasing when the contents of GMs increased to 30wt% and 40wt%. Furthermore, based on the interpretations above, we can assume that the thermal conductivity of the synthetic foam would increase continuously if much more microspheres were added. But back to the fact we could not add the microspheres without limit for there must be a maximum amount of filler which can be fully wetted by the resin matrix in this system. All in all, the best experimental conditions occurred when 20wt% of the hollow glass microspheres were added to the phenolic resin and the thermal conductivity of the composite was improved by ∼56.7% compared with the neat resin.

## 4 Conclusion

Phenolic resin based composite syntactic foams were successfully fabricated by mechanical mixing with different amounts of GMs. Tensile strength decreased with the increasing microsphere content, while the corresponding tensile modulus increased. Overall compression strength of the syntactic foams decreased, while the strength and modulus for syntactic foams with 10wt%, 20wt%, 30wt% microspheres are similar to each other. All these changes in mechanical properties could be attributed to the microspheres acting as voids and weaken the structure. The thermal conductivity of the sample with 20wt% microspheres reached a minimum value of approximately 0.129 W (m·K)^−1^ which is ∼46.7% lower than the neat one’s [0.298 W (m·K)^−1^]. Compared with traditional core materials, the polymeric foam is affordable and easily prepared with low cost, excellent thermal insulate performance as well as proper mechanical properties. It is ideal candidates for various engineering applications such as construction industry, aerospace industry and so on.

## Data Availability

The original contributions presented in the study are included in the article/Supplementary Material, further inquiries can be directed to the corresponding author.
